# Deletion of ACC Deaminase in Symbionts Converts the Host Plant From Water Waster to Water Saver

**DOI:** 10.1111/pce.15265

**Published:** 2024-11-07

**Authors:** Katharina Hecht, George A. Kowalchuk, R. Ford Denison, Ansgar Kahmen, Wu Xiong, Alexandre Jousset, Mohammadhossein Ravanbakhsh

**Affiliations:** ^1^ Institute of Environmental Biology, Ecology and Biodiversity Utrecht University Utrecht The Netherlands; ^2^ Department of Ecology, Evolution, and Behavior University of Minnesota – Twin Cities St. Paul Minnesota USA; ^3^ Department of Environmental Sciences, Physiological Plant Ecology Group The University of Basel Basel Switzerland; ^4^ Jiangsu Provincial Key Lab of Solid Organic Waste Utilization, Jiangsu Collaborative Innovation Center of Solid Organic Wastes, Educational Ministry Engineering Center of Resource‐Saving Fertilizers Nanjing Agricultural University Nanjing China

**Keywords:** ACC deaminase, drought, ethylene, plant‐associated bacteria, plant hormone

## Abstract

Increasing drought events coupled with dwindling water reserves threaten global food production and security. This issue is exacerbated by the use of crops that overconsume water, undermining yield. We show here that microorganisms naturally associated with plant roots can undermine efficient water use, whereas modified bacteria can enhance it. We demonstrate that microbe‐encoded genes shape drought tolerance, likely by modulating plant hormonal balance. Specifically, we built a minimal holobiont out of *Arabidopsis thaliana* and either the bacterium *Pseudomonas putida* UW4 or its isogenic AcdS^−^ mutant, lacking the enzyme ACC deaminase. This enzyme breaks down the precursor of ethylene, a key regulator in plant response to drought. This single mutation profoundly affected plant physiology and shifted the plant from a ‘water‐spender’ (with more growth under well‐watered conditions) to a ‘water‐spender’ phenotype. Under drought, plants associated with wild‐type bacteria consumed soil water faster, leading to a shorter period of growth followed by death. In contrast, plants associated with the AcdS^−^ mutant managed to maintain growth by reducing water consumption via stomatal closure, thus conserving soil water. This allowed plants to survive severe water deficiency. We conclude that plant‐associated bacteria can modulate plant water use strategies, opening possibilities to engineer water‐savvy crop‐production systems.

## Summary statement

In this study, we show that altering a single gene in plant‐associated bacteria can modulate plant ethylene balance and improve drought tolerance. Deleting the ACC deaminase gene shifts the host plant's behaviour from excessive water use to a more conservative water‐saving strategy.

## 1 Introduction

Drought events are becoming increasingly frequent and severe, resulting in drastic crop yield reductions in affected areas, thereby threatening global food security (He et al. 2019; Mehrabi and Ramankutty 2019; Ray, Fares, and Risch 2018). Plants have evolved various drought responses, which can be classified into two general strategies, water‐spender and water‐saver (Urban et al. 2017). The water‐spender strategy may maximize individual fitness by a prodigal use of the water available. Plants following this strategy tend to maintain stomatal transpiration while reducing the leaf water potential and increasing the uptake of water and nutrients from the soil (Bueno et al. 2019; Guehl and Aussenac 1987; Lo Gullo and Salleo 1988). The water‐saver strategy relies on limiting water use by restraining water uptake, transport through the plant, and evaporation, through reduced leaf area (in drought‐deciduous plants) or early stomatal closure (Bueno et al. 2019; Guehl and Aussenac 1987; Lo Gullo and Salleo 1988). This strategy could allow plants to cope much longer with a reduced water supply. Limiting water use, especially when the vapour‐pressure deficit is high, may also increase water use efficiency (WUE; Denison 2015; Schoppach et al. 2017).

In the context of plant communities, water‐use strategy may be explained from a game theory perspective. Water is here a public good that can be either used cooperatively, maximizing group‐level growth per unit of available water, or selfishly, potentially maximizing individual fitness at the cost of the neighbouring plants (Bueno et al. 2019; Zea‐Cabrera et al. 2006). In mixed plant communities, like those in which crop ancestors evolved, a selfish water‐spender may gain a fitness advantage by consuming the limited water available, at the expense of any cooperative, water‐saver neighbours. As spenders come to dominate, however, they reduce population‐level WUE by keeping stomata open even when water demand in the atmosphere and thus transpiration is high (e.g., on hot, dry afternoons). In an agricultural setting with fields comprised of nearly identical plants, cooperative water use could maximize yields under water limitation. The water‐spender strategy may be consistent with plant‐community productivity during short‐term or mild drought (Nakhforoosh et al. 2016). Under severe water limitation, however, the more individuals follow a spender strategy, the sooner the local water supply in the soil gets exhausted, leading to plant damage or death.

Plant drought tolerance is an interactive result of a plant's own genes as well as those encoded in its associated microorganisms (Marulanda, Barea, and Azcón 2009; Naylor and Coleman‐Derr 2018; Ulrich et al. 2019). Plants typically live in association with billions of bacteria, fungi, and protists (Hassani, Durán, and Hacquard 2018). Together, these microorganisms harbour a functional gene pool several orders of magnitude more diverse than the repertoire encoded in the plant genome. Microbial genes may influence plant stress responses, such as by providing protective molecules that the plant is unable to produce itself. In the context of drought tolerance, microbially produced molecules, such as exopolysaccharides, can protect plant roots against damage due to dehydration (Marasco et al. 2022). Associated microbes may also alter key plant hormonal pathways involved in drought tolerance, such as auxin, abscisic acid (ABA) (Singh and Roychoudhury 2023), or ethylene signalling (Egamberdieva et al. 2017; Glick et al. 2007). Altering these hormonal pathways can directly modify plant phenotypes or influence them indirectly by affecting the cross‐talk between hormones, for example between ABA and ethylene (Caarls et al. 2017; Wilkinson and Davies 2010).

We focus on ethylene signalling as a target hormone. Ethylene is a central plant hormone regulating, among other things, plant adaptation to abiotic stresses such as drought (Naing et al. 2022). In the production of ethylene, a crucial step involves the synthesis of its precursor, 1‐aminocyclopropane‐1 carboxylic acid (ACC), catalyzed by the enzyme ACC synthase (ACS). When stress is detected, ACS facilitates the conversion of ACC into ethylene, a reaction further catalyzed by the enzyme ACC oxidase (ACO). High plant ethylene levels, a consequence of this process, are consistently associated with increased stress resistance (Etesami et al. 2020; Ravanbakhsh 2018; Shekhawat et al. 2022). Ethylene signalling is an interesting model to study microbial effects on plant physiology: It is well characterized, and both plant and bacterial genes involved in its regulation are well described (Ravanbakhsh 2018). Ethylene‐modulating microorganisms are diverse, and they might be transmitted vertically, that is, in seeds (Bergna et al. 2018) or horizontally (Weingart, Völksch, and Ullrich 1999), making ethylene balance a product of interactions between plants and their associated microbiome (Ravanbakhsh et al. 2018). By cleaving the ethylene precursor ACC (1‐aminocyclopropane‐1‐carboxylate), some bacteria can obtain nitrogen from plants, while at the same time, they may reduce ethylene concentrations in the plant (Ravanbakhsh et al. 2018). Given the crucial role of ethylene as a trigger in plant resistance to drought, we expect that bacteria altering ethylene production may have profound impacts on plant responses to water scarcity. Could appropriate plant‐microbe combinations promote water‐use strategies that improve plant‐community performance under drought?

In this study, we built a minimal holobiont model composed of one plant and one bacterial strain (Ravanbakhsh, Kowalchuk, and Jousset 2021). As a model bacterium, we used the strain *Pseudomonas putida* UW4, which is able to colonize plant roots and is known to catabolize the ethylene precursor ACC (1‐aminocyclopropane‐1‐carboxylate) and use it as a source of nitrogen (Penrose, Moffatt, and Glick 2001). The spatial structure of this process remains to be explored. Previous studies have shown that reducing ACC via the action of plant‐associated bacteria causes lower ethylene concentrations in plants, which can improve plant growth under benign conditions but which can seriously impair the plant's ability to respond to abiotic stressors such as flooding or heavy metal exposure (Ravanbakhsh, Kowalchuk, and Jousset 2019b). To assess the importance of microbial effects on ethylene signalling and water use, we inoculated plants with either wild‐type *Pseudomonas putida* UW4 or an isogenic mutant strain in which the gene responsible for ACC degradation was deactivated. Plants without bacterial inoculation were used as an additional control. We exposed the plants to increasing levels of water deficiency and assessed plant responses at a physiological level (relative stomatal opening, root development). In a previous study employing the same experimental design in terms of plant and bacteria models, we observed a reduction in ethylene levels by bacteria both in non‐stress conditions and under various abiotic stresses. Building upon this knowledge, we hypothesized that (1) unmanipulated Arabidopsis responds to water stress by adopting a more water‐saving phenotype, (2) bacteria that impair ethylene signalling hinder this response, reducing plant adaptation to water deficiency, and (3) less‐conservative phenotypes will exhibit impaired survival under prolonged drought. To the extent that the holobiont containing wild‐type bacteria (WT) interferes with ethylene signalling, we predicted that plants would fail to acclimate to high levels of drought stress by limiting transpiration (Ravanbakhsh et al. 2017). In contrast, plants associated with the mutant bacteria were expected to maintain ethylene signalling, leading to more‐conservative water use and therefore improved survival under drought. We will further discuss how targeted holobiont engineering might present new opportunities for improving plant drought resistance.

## 2 Materials and Methods

We built a minimal holobiont constituted of one plant and one bacterial strain (Ravanbakhsh, Kowalchuk, and Jousset 2021). This model is conceived to port classical genetics studies to a multiorganism level. The new model modulates plant phenotype as the product of an intimate interaction between plants and their associated microorganisms (Ravanbakhsh, Kowalchuk, and Jousset 2021). If we hold plant genotype constant, plant phenotype becomes part of the extended phenotype of the bacteria (Dawkins 1982).

### 2.1 Model Plant

We used *Arabidopsis thaliana* (L.) Heynh., ecotype Columbia (hereafter referred to as ‘Col‐0’) as a C3‐photosynthetic plant model for our experiments (Hoover, Duniway, and Belnap 2015).

### 2.2 Model Bacterial Strains

Wild‐type *Pseudomonas putida* UW4 (hereafter referred to as ‘WT’) was used as a model root‐colonizing bacterium (Duan et al. 2013). It has been extensively used in the study of plant physiology under diverse stress conditions. This bacterium is representative of the wide range of root‐associated bacteria producing the enzyme ACC deaminase. This enzyme allows bacteria to consume the ethylene precursor ACC as a carbon and nitrogen source (Glick, Penrose, and Li^1998^; Stearns et al. 2012). To assess the effect of bacterial degradation of ACC on the plant phenotype, we used a genetically engineered isogenic mutant (hereafter referred to as ‘AcdS^−^ mutant’) lacking the ACC deaminase gene (*acdS* ^*−*^). The bacterial mutant (AcdS^−^) was obtained by inserting a tetracycline resistance gene into the ACC deaminase gene coding region (Li et al. 2000). Both strains were kindly provided by Prof. Bernard Glick, Department of Biology, University of Waterloo, Canada. Bacterial strains were kept as frozen stocks at −80°C. Before experiments, one single colony was grown overnight on 30 g L^−1^ Tryptic soy broth (TSB) supplemented with 100 µg mL^−1^ Ampicillin (for the wild‐type bacteria) or TSB supplemented with 50 μg ml^−1^ tetracycline for AcdS^−^ mutant. Tryptic soy broth medium consisted of tryptone (17 g L^−1^), neutralized soya peptone (3 g L^−1^), NaCl (5 g L^−1^), dipotassium phosphate (K_2_HPO_4_) (2.5 g L^−1^), and glucose (2.5 g L^−1^). Bacteria were harvested by centrifugation (6000*g*, 10 min), washed three times in 10 mM MgSO_4_, and adjusted to a density of 10^8^ cells mL^−1^ before inoculation. Fifty microliters of the bacteria suspension was added to the base of each plant (in the soil) on Day 25 after sowing. For the uninoculated control plants, 50 µL of MgSO₄ solution alone was added in place of the bacteria suspension. We evaluated the density of wild‐type and AcdS^−^ mutant strains on plant roots on Day 35 (Supporting Information S1: Figure S1). Roots were gently shaken to remove soil, and bacteria were extracted by shaking in 10 mM MgSO₄, followed by sonication and vortexing. Bacterial counts were obtained by serial dilution on selective media: wild‐type on DF agar with ACC as the nitrogen source and ampicillin, and the AcdS^−^ mutant on DF agar with (NH₄)₂SO₄ and tetracycline.

### 2.3 Experimental Design

Plants given three different bacteria treatments were exposed to a range of drought conditions. We grew a total of 63 plants, of which 21 plants were inoculated with the wild‐type bacterial (WT) strain, another 21 plants were inoculated with the AcdS^−^ mutant strain, and 21 control plants were without bacterial inoculation. We set up six levels of drought plus one well‐watered control. We first measured the soil moisture content and estimated the soil field capacity (Ray, Fares, and Risch 2018; Walker 1989) (Supporting Information S1: Text 1). This soil‐water level (80% of the soil moisture content in saturated soil or 23 mL water for 120 g soil in each pot) was considered as a nondrought condition (control). Nine plants (three plants inoculated with AcdS^−^ mutant bacteria, three plants inoculated with the wild‐type bacteria (WT), and three plants without explicit bacteria treatment) were used for each water treatment. We manipulated the watering frequency to adjust the target soil water content for different drought levels. Water availability was manipulated by spacing the water application of 23 mL filtered and deionized water (Milli‐Q water) per plant every 12 h for well‐watered control (D0), versus every 24, 36, and 48 h for mild drought treatments (D1, D2, and D3, respectively), and every 3, 4, and 18 days for severe drought treatments (D4, D5, and D6, respectively) analogous to decreasing frequency of rain in the field. Drought manipulation took place for 18 days. The rationale behind choosing the water volume (instead of weighting the pots) was to address the complexity of variations in pot biomass due to the potential effects of bacterial treatments on plant biomass. Using pot weight without considering the biomass variation could be misleading, especially when the plant biomass is a significant fraction of the total weight.

### 2.4 Set‐Up of the Pot Experiment

*Arabidopsis thaliana* seeds were surface‐sterilized by chlorine gas as follows: seeds were placed in an open microcentrifuge tube and incubated next to a 250 mL glass beaker with 100 mL NaClO, and 3.2 mL concentrated HCl for 4 h in a 7 L airtight desiccator jar (Clough and Bent 1998). Immediately after that, seeds were sown on autoclaved potting soil (Potgrond Holland B.V.) mixed with sand and perlite in a 1:1:1 ratio. Seeds were then covered with a dark plastic foil to avoid exposure to light and vernalized at 4°C in a cold room for 4 days. In the next step, plant seeds were transferred to a walk‐in growth chamber (20°C; 70% relative humidity, 12 h photoperiod, 200 µmol m^−2^ s^−1^ PPFD) and covered with a transparent lid for the first 2 days to avoid desiccation. Sixty‐three (63) seedlings were selected based on the homogeneity of their developmental stage on Day 16 (4‐leaf stage plants). Seedlings were transplanted to individual 70 mL pots containing a mixture (ratio 3:3:2) of sand, perlite, and potting soil (pasteurized at 90°C), enriched with Osmocote (10 mg/L potting soil). All pots were drenched with 20 mL of half‐strength autoclaved Hoagland solution (Hoagland and Arnon 1950). Plant rhizosphere was inoculated with bacterial strains on Day 25 (by adding the bacterial suspension on the bottom of the plant stem in soil) and maintained under well‐watered conditions until Day 35 after germination. Our experimental design adhered to our standard operating procedure (Ravanbakhsh, Kowalchuk, and Jousset 2021), with the bacteria allowed to colonize the rhizosphere for 10 days until Day 35, resulting in a significant reduction in ethylene levels. Ethylene level was measured on Day 35 and before the onset of drought stress (next section). Drought treatments were initiated on Day 35 and continued for 18 days. Pots were randomized daily to minimize potential bias. Due to the destructive nature of ethylene measurement methods, ethylene measurement was not performed in this study under different levels of drought.

### 2.5 Ethylene Measurement

We measured ethylene production in plant aerial parts on Day 35. While limited amounts of ethylene can also be synthesized in roots, measuring ethylene production in the shoot is a standard and validated method to estimate ethylene concentrations in plants (Cristescu et al. 2013). To do so, shoots were carefully separated from the roots with a razor blade, and aerial parts were placed in serum vials (50 mL). After 2 h, a 1 mL gas sample was taken from each vial and injected into a Chrompack Packard gas chromatograph model 438A with a Poropack Q column (length 100 cm, packed to 0.34 g cm^−3^) (Chrompack) at 60°C to measure ethylene production. Ethylene concentrations were expressed in pL g^−1^ FW h^−1^ (Voesenek et al. 2003).

### 2.6 Water Consumption Monitoring

Water consumption of each plant was estimated based on the weight difference between supplied and remaining water before rewatering the plants and expressed as mL H_2_O day^−1^. We covered the soil surface with aluminium foil to prevent water evaporation.

### 2.7 Relative Stomatal Opening

We used the silicon polymer impression technique (Johnson 2007) for relative stomatal opening measurement. One representative leaf (leaf no. 4) of each plant was cut and directly transferred to a prepared dental polymer paste (EXAFINE; GC Corporation) on Day 48 of the experiment (17 days after beginning of drought). After drying the imprint of each leaf for approximately 15 min, the leaves were carefully removed from the polymer paste, and imprints were covered with a thin layer of transparent nail polish. After drying, the nail polish layer was carefully removed with tweezers and placed on a microscope slide. The slide was covered with a slide coverslip and sealed using transparent nail polish. Stomata were counted on an Olympus BX50 WI inverted microscope with a × 20 objective. The number of open and closed stomata was counted manually on four randomly chosen leaf areas each of 0.143 mm^2^. We used the fraction of open stomata out of total stomata to estimate ‘relative stomatal opening’.

### 2.8 Harvest and Biomass Analysis

On Day 54, roots were separated from shoots with a razor blade, and shoot and root fresh‐weight were measured. The shoot and root dry weight of plants were analysed after drying in the oven (at 70°C until weight remained constant). The root‐shoot ratio was calculated for all plants based on plant dry weight.

### 2.9 Plant Drought Tolerance Index

We recorded leaf necrosis of *Arabidopsis thaliana* plants in all drought intensity levels at the end of the experiment. Plant rosettes were scanned into a digital format using an Epson Expression 10000XL scanner (Seiko Epson Corporation) and saved as 1200 dpi, colour, uncompressed TIFF files. Images were then digitally analysed for green and wilted leaf areas using ImageJ (bundled with 64‐bit Java 1.8.0; National Institutes of Health), using a threshold‐based pixel count measurement. This method has previously been shown to provide reliable measurements in the analysis of leaf area, shape, and diversity (Sack and Frole 2006). A ‘drought‐tolerance’ (or drought‐avoidance) index was calculated based on the diameter of the green area measured for each drought level as a proportion of the diameter of the green area in the well‐watered, uninoculated treatment. Plants were considered dead when all leaves and roots were dried and brittle (Noodén and Penney 2001).

### 2.10 WUE

Together with the fraction of open stomata and the root‐shoot ratio, we measured one form of proxy for WUE, specifically the ratio between final plant dry weight (mg) (including growth before the water treatments) and total water consumption by plants (mL) over 18 days (Medrano et al. 2015).

$$\mathrm{WUE}=\frac{\mathrm{Plant}\;\mathrm{DW}\;\left(\mathrm{mg}\right)}{\mathrm{Consumed}\;\mathrm{water}\;\left(\mathrm{mL}\right)}.$$

### 2.11 δ^13^ *C* Isotope Analysis

This method relies on the natural abundance of δ^12^ *C* and δ^13^ *C* isotopes in plant tissues. Plants naturally absorb carbon dioxide (CO_2_) from the atmosphere, which contains both δ^13^ *C* and δ^13^ *C* isotopes. The ratio of these isotopes in plant tissues can vary based on environmental conditions, particularly water availability. In this study, the stable‐isotope ratio (δ^13^ *C* values) of plant shoots was analysed as a quantitative measure of the stress experienced by the plants in different drought intensities. δ^13^ *C* values in the leaf strongly correlate with the intrinsic WUE (i.e., the ratio of net photosynthesis over transpiration). WUE typically increases when plants have to conserve water and stomata close, making δ^13^ *C* values a useful physiological drought stress indicator (Arndt and Wanek 2002a; Farquhar, O'Leary, and Berry 1982; Moghaddam et al. 2013; Pate 2001; Stewart et al. 1995).

For δ^13^ *C* measurements, dried plant samples were prepared by grinding the shoots to a fine powder using a ball mill. The isotope amounts in each sample were analysed using an elemental analyzer (One Thermo Flash 2000, Thermo Fisher Scientific Inc. Germany).

δ^13^ *C* was calculated as the δ^13^ *C*/δ^12^ *C* ratio offset for both raw and drift‐corrected delta from two in‐house reference standards analysed three times throughout the sequence.

$$\mathrm{Offset}=\left(\frac{R_{\mathrm{measured}}}{R_{\mathrm{calibrated}-1}}\right)\times 1000=\left(\frac{\left(delta_{\mathrm{measured}}+1000\right)}{\left(delta_{\mathrm{calibrated}}+1000\right)}-1\right)\times 1000,$$

$$\delta^{13}C\left(‰\right)=\left(\frac{R\mathrm{sample}}{R\mathrm{calibrated}\;-\;1}\right)\times 1000.$$

where *R*_sample_ is the δ^13^ *C*/δ^12^ *C* ratio and *R*_calibrated_ is the calibrated laboratory standard for reference.

### 2.12 Statistical Analyses

We employed a regression model that incorporates a continuous independent variable (continuous predictor (watering frequency [day^−1^]) and plant traits (e.g., shoot biomass, stomata closure) as a continuous dependent variable for each of the inoculation treatments (WT, AcdS^−^, and uninoculated). This model allows us to use fewer replicates (three replicates per level of drought) while effectively capturing trends and relationships across the gradient of treatments. Descriptive statistics of residuals (Supporting Information S1: Table S1) showed that the mean residuals for all traits are centred around zero, indicating that the models capture the central trends well. Skewness and kurtosis values were calculated to confirm the normality of the residuals (Supporting Information S1: Table S2). While some traits, such as shoot dry weight and δ¹³*C*, exhibited lower *R*² values, suggesting potential biological variability or interaction effects, the overall trends remain robust across other traits. To determine the most suitable regression models for our analysis, we used the Akaike Information Criteria (AIC) as a guiding principle (Supporting Information S1: Table S3). The AIC is a statistical metric that strikes a balance between model goodness of fit and complexity. By selecting models with lower AIC values, we prioritize those that offer the best trade‐off between accurately capturing the data patterns and avoiding excessive complexity.

**Figure 1:**
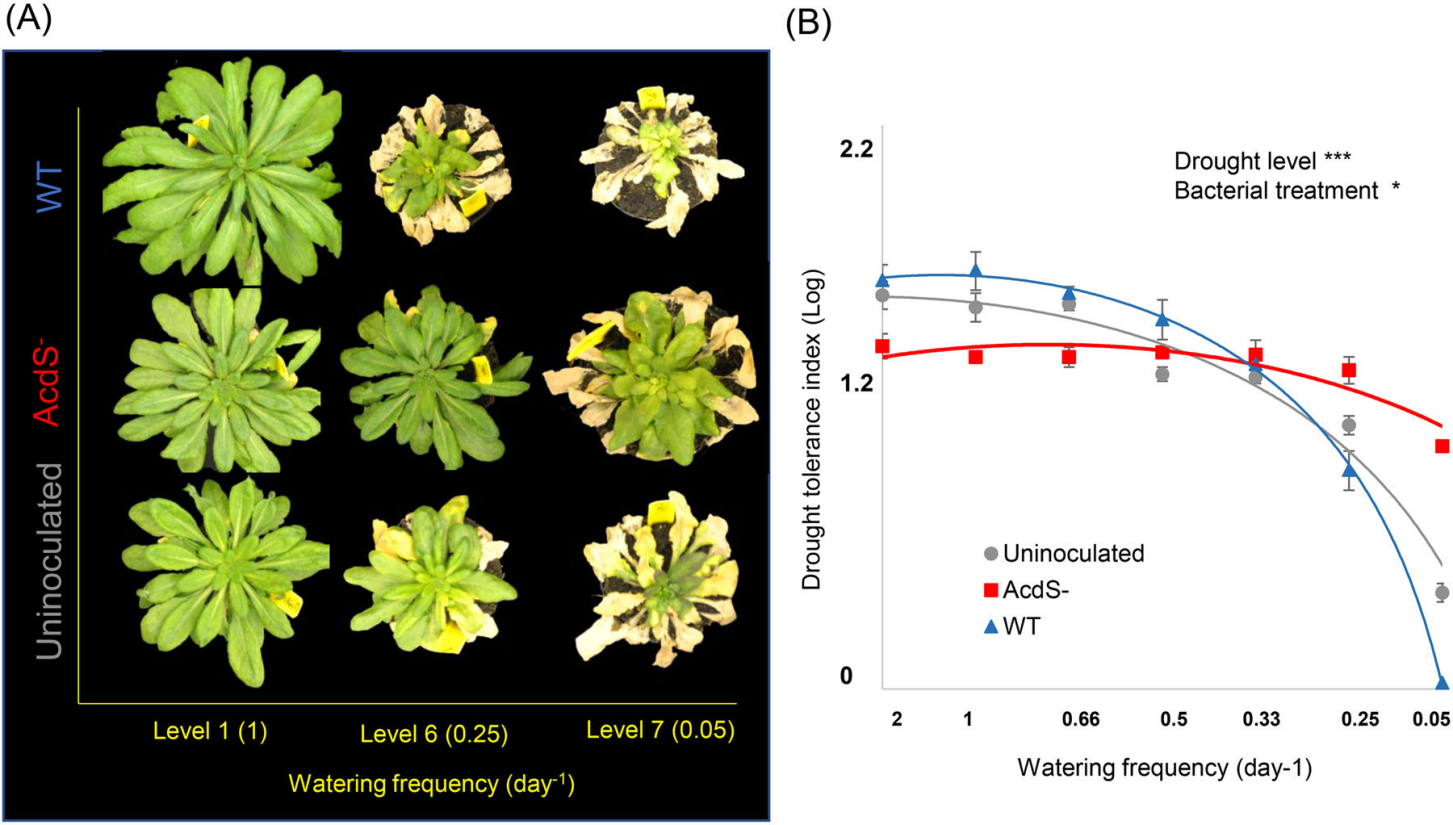
Effects of bacterial treatments on plant drought tolerance. (A) Comparison of representative *A. thaliana* Col‐0 rosettes grown alone or in association with wild‐type *P. putida* UW4 (WT) producing ACC (1‐aminocyclopropane‐1‐carboxylic acid) deaminase or its isogenic mutant lacking ACC deaminase (AcdS^‐^). Drought intensity was manipulated by reducing the irrigation frequency, ranging from well‐watered condition (Control plants watered every 12 h with 23 mL water) to mild drought (D1, D2, and D3, watered every 24, 36, and 48 h, respectively), to severe drought treatments (D4, D5, and D6 watered every 3, 4, and 18 days, respectively). (B) Drought stress visual index in the *A. thaliana* Col‐0 plant under different drought intensities. The drought tolerance index was defined as the percent of an average diameter of green plant rosette in each drought level divided by the green plant rosette in the well‐watered treatment (in no‐bacterial control) at a logarithmic scale. Details of the data analysis are provided in Supporting Information S1: Tables S3 and S5. Error bars show ± SE.

We performed ANOVA to assess the overall significance of the model, and later post‐hoc comparisons to assess the specific differences between the treatment groups (three levels: uninoculated control, wild‐type bacteria (WT) and ACC deaminase‐deficient mutant (AcdS^−^) on plant morphology and drought tolerance. Due to a high mortality rate in the highest drought intensity (lowest‐frequency watering level, D6 in Figure 1), this treatment was not included in the analyses of morphology and stress tolerance. Root‐to‐shoot ratio and stomatal data were transformed using Log_10_ before ANOVA analyses. We also compared linear, quadratic, and cubic models to account for potential nonlinear relationships, such as those observed in the root‐to‐shoot ratio and WUE. Statistical analyses were carried out in SPSS V.22 (IBM Corp.) and R (package MASS and POA).

## 3 Results

### 3.1 Leaf Necrosis and Plant Survival

We recorded leaf necrosis of *Arabidopsis thaliana* plants for all drought‐intensity levels. In the well‐watered treatment, plants inoculated with the wild‐type strain had significantly larger green plant rosette diameter (*F*_2, 54_ = 3.92, *p* = 0.02) than uninoculated or AcdS^−^ plants (Figure 1A,B). However, plants inoculated with the AcdS^−^ mutant bacteria showed significantly larger green plant rosette diameter under drought condition (Table 1) (Figure 1A,B) compared with wild‐type bacterial (WT) inoculation and uninoculated treatments. The plants inoculated with AcdS^−^mutant bacteria also showed a substantially higher survival rate (*F*_1, 23_ = 15.42, *p* < 0.001) at the three higher levels of drought (D3, D4, and D5 with watered at intervals greater than 72 h) compared with wild‐type bacteria (WT) and uninoculated treatments.

**Table 1:** ANOVA table summarizing the results of multiple linear regression model using water gradient as a continuous independent variable (continuous predictor, ln(watering frequency [day^−1^]) and plant traits (e.g., Shoot biomass, stomata closure) as a continuous dependent variable.

Dependent variables	Source	*df*	*F*‐value	*p* Value
Plant drought tolerance^a^	Corrected model	7	8.05	**< 0.001**
	Bacterial treatments	2	3.92	**0.02**
	Drought level	5	9.71	**< 0.001**
	Error	54		
Shoot dry weight	Corrected model	7	3.57	**0.001**
	Bacterial treatments	2	0.58	0.12
	Drought level	5	4.77	0.15
	Error	54		
Root dry weight	Corrected model	7	3.63	**< 0.001**
	Bacterial treatments	2	9.64	**< 0.001**
	Drought level	5	1.23	**0.04**
	Error	54		
Root/shoot ratio (Log)	Corrected model	7	13.16	**< 0.001**
	Bacterial treatments	2	27.11	**< 0.001**
	Drought level	5	7.58	**< 0.001**
	Error	54		
Water consumption	Corrected model	7	10.83	**< 0.001**
	Bacterial treatments	2	16.04	**< 0.001**
	Drought level	5	8.74	**< 0.001**
	Error	54		
Relative stomatal opening (log)	Corrected model	7	11.97	**< 0.001**
	Bacterial treatments	2	4.17	**< 0.001**
	Drought level	5	15.10	**< 0.001**
	Error	54		
Water use efficiency	Corrected model	7	1.98	**0.05**
	Bacterial treatments	2	4.45	**0.01**
	Drought level	5	0.99	0.12
	Error	54		
δ^13^ *C*	Corrected model	7	11.23	**0.02**
	Bacterial treatments	2	31.61	**0.04**
	Drought level	5	3.08	**0.002**
	Error	54		

*Note:* Bacterial inoculation (uninoculated, Wild‐type bacteria (WT) interfering with ethylene signalling or their isogenic *AcdS‐* mutant). Bold values indicate statistically significant results (*p* < 0.05).

^a^ We recorded leaf necrosis and calculated the percentage of wilted leaf area out of the total leaf area (within each plant rosette) and considered it as a plant drought tolerance.

### 3.2 Plant Ethylene, Dry Weight, and Shoot‐To‐Root Ratio

**Figure 2:**
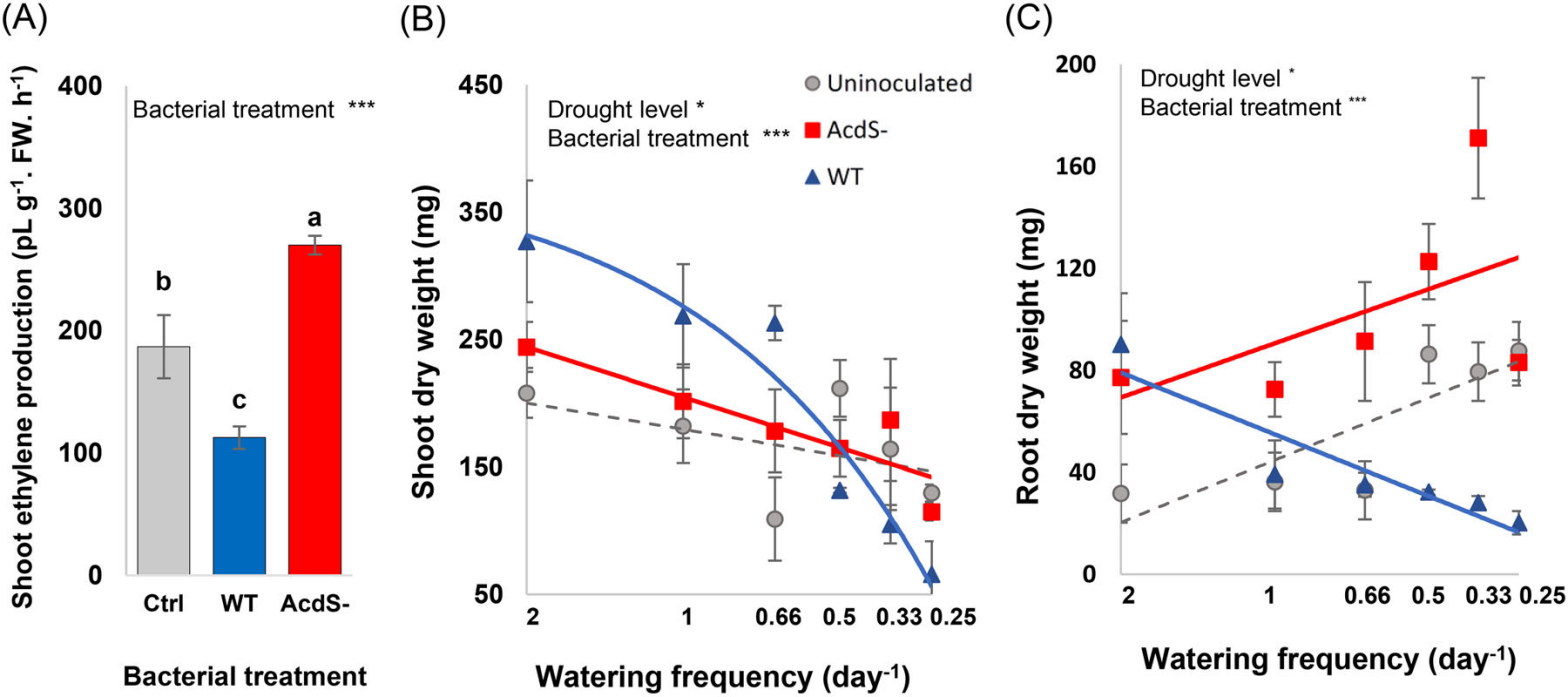
Effects of bacterial treatments on plant ethylene and plant weight. (A) Shoot ethylene production (pL g^−1^ FW h^−1^) in *Arabidopsis thaliana* Col‐0 plants inoculated with different bacterial treatments on Day 35 (10 days after inoculation with bacteria). The treatments include ACC deaminase‐producing wild‐type *P. putida* UW4 (WT), ACC deaminase‐deficient mutant (AcdS^‐^), and an uninoculated control (Ctrl) with no inoculation. Different letters show significant differences among treatments based on the Tukey post hoc test. Error bars represent standard error. Interactive effects of drought intensity (indexed by watering frequency) and bacteria inoculation (ACC deaminase producing wild‐type *P. putida* UW4 (WT), ACC deaminase‐deficient mutant (AcdS^‐^), and an uninoculated control) on (B) shoot dry weight, and (C) root dry weight of *A. thaliana* Col‐0. Drought intensity (indexed as the watering frequency on the *x*‐axis) was manipulated by reducing the irrigation frequency, ranging from well‐watered condition (Control plants watered every 12 h with 23 mL water) to mild drought (D1, D2, and D3, watered every 24, 36, and 48 h, respectively), to severe drought treatments (D4 and D5 watered every 3 and 4 days, respectively). Trendlines are shown in grey for uninoculated plants, in solid blue line for plants inoculated with wild‐type bacteria (WT), and solid red line for plants inoculated with AcdS^‐^ mutant bacteria. Where the model fit was not significant (Supporting Information S1: Table S3), the full lines changed to dashed lines indicating nonsignificant fits. Error bars show ± SE. Asterisks are significantly different according to a GLM. Details of the data analysis are provided in Supporting Information S1: Tables S3 and S5. (***p < 0.0001, **p < 0.001, *p < 0.05). [Color figure can be viewed at wileyonlinelibrary.com]

Ethylene concentrations varied significantly among different bacterial treatments (Figure 2A). The plants inoculated with AcdS^−^ bacteria showed higher ethylene concentration than the uninoculated control, which had more ethylene than plants inoculated with wild‐type bacteria (Figure 2A). Thus, the absence of a single acdS gene reversed the effects of bacteria on this plant phenotype (ethylene). We evaluated the bacterial density on the roots of the plants inoculated with wild‐type or AcdS^−^ bacterial mutant. The results showed that the absence of the acdS gene did not impact bacterial density on the roots (Supporting Information S1: Figure S1). Additionally, the colonization rate of the wild‐type strain was negatively correlated with shoot ethylene production.

Increasing drought intensity significantly reduced shoot dry weight (Figure 2B, Table 1, main effect of Drought and Bacterial treatments). Plants associated with ACC deaminase‐producing wild‐type bacteria (WT) produced higher shoot dry weight in well‐watered conditions than plants associated with the AcdS^−^ mutant and uninoculated plants (Figure 2B, Supporting Information S1: Table S4, main effect of Bacterial treatments on shoot dry weight) but severe drought apparently affected the WT treatment more (Figure 2B).

A significant difference in root dry weight was observed between bacterial treatments (Figure 2C, Table 1, the main effect of bacterial treatments on root dry weight). The effect of bacteria depended further on water availability (Table 1, significant interactive effects of Bacterial treatments and Drought). In the well‐watered condition, both bacterial strains stimulated root growth. The response is similar for both strains, suggesting that this effect is not related to ethylene signalling. The relevance of ethylene signalling apparently became stronger under drought condition. While plants inoculated with the AcdS^−^ mutant produced more root dry weight with increasing drought intensity, plants inoculated with the wild‐type bacteria (WT) invested significantly less in root biomass production as drought intensity increased (Figure 2C, Table 1, significant interactive effects of Bacterial treatments and Drought).

**Figure 3:**
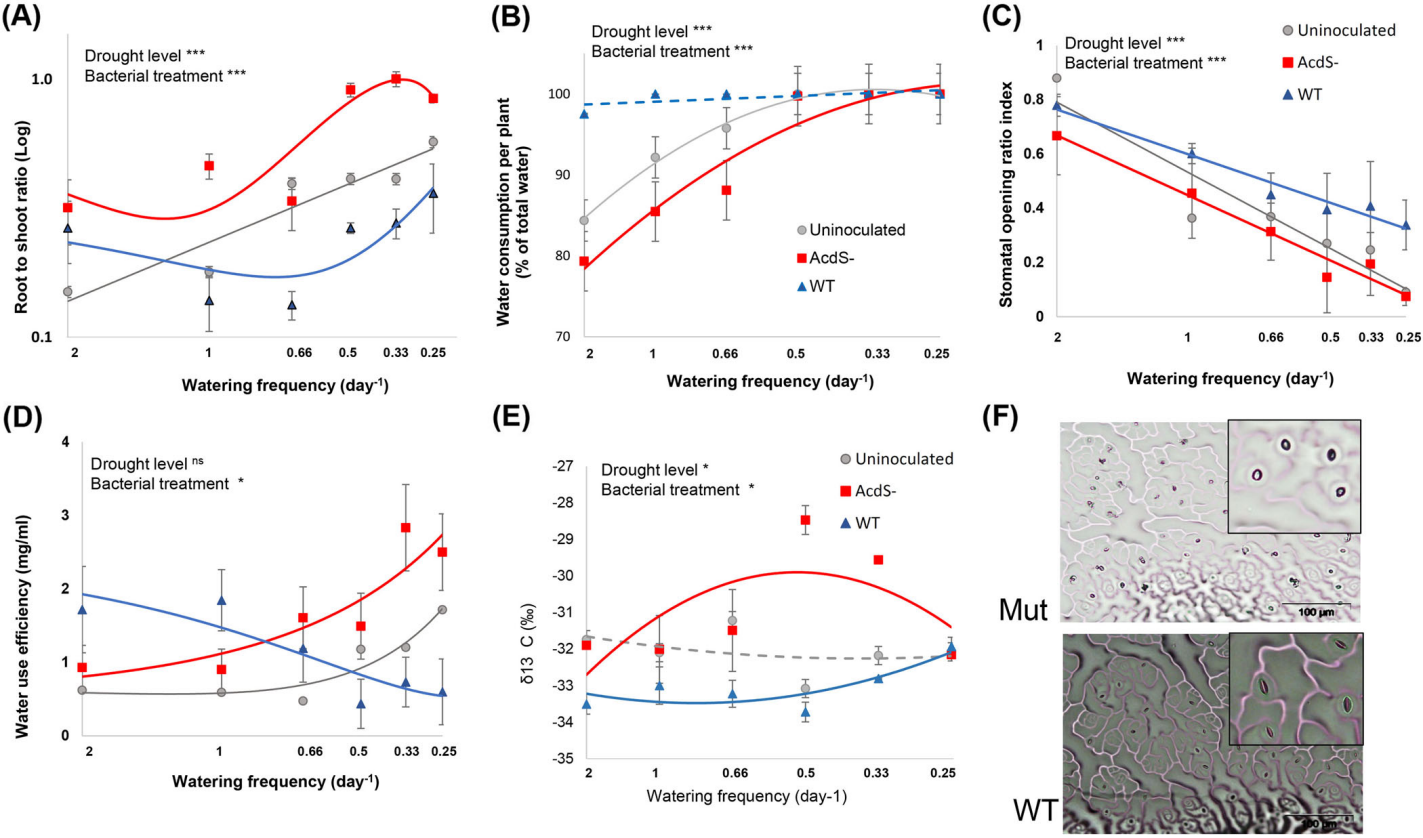
Effects of bacterial treatments on drought tolerance factors. Interactive effects of drought intensity (indexed by watering frequency) and bacteria inoculation (ACC deaminase producing wild‐type *P. putida* UW4 (WT), ACC deaminase‐deficient mutant (AcdS^‐^), and an uninoculated control) on (A) root‐shoot ratio, (B) water consumption per plants, (C) relative stomatal opening, (D) water use efficiency (WUE), and (E) stable isotope ratio δ^13^ *C* in plant shoots of *A. thaliana* Col‐0. Drought intensity (indexed as the watering frequency on the *x*‐axis) was manipulated by reducing the irrigation frequency, ranging from well‐watered condition (Control plants watered every 12 h with 23 mL water) to mild drought (D1, D2, and D3, watered every 24, 36, and 48 h, respectively), to severe drought treatments (D4 and D5 watered every 3 and 4 days, respectively). Trendlines are shown in grey for uninoculated plants, in solid blue line for plants inoculated with wild‐type bacteria (WT), and solid red line for plants inoculated with AcdS^‐^ mutant bacteria. Where the model fit was not significant (Supporting Information S1: Table S3), the full lines changed to dashed lines indicate nonsignificant fits. For the log‐transformed values of root to shoot ratio (A) total water fits with a linear model. Error bars show ± SE. Significant differences according to a GLM is depicted by asterisks (***p < 0.0001, **p < 0.001, *p < 0.05). Details of the data analysis are provided in Supporting Information S1: Table S3. (F) Shows microscopic images of closed (e.g., blue circles) and open (e.g., red circles) stomata on randomly selected leaf areas (each with a surface area of 0.143 mm^2^) of *A. thaliana* inoculated with the wild‐type bacteria (WT) and ACC deaminase‐deficient mutant (AcdS^‐^) under drought level 4 (log of watering level: 2.26). Images were captured with an Olympus BX50 WI inverted microscope with a × 20 objective. [Color figure can be viewed at wileyonlinelibrary.com]

The effects of drought on the plants (and potential differences between WT, acdS^−^, and uninoculated treatments due to ethylene modification) are more accurately reflected in the root‐to‐shoot ratio rather than in shoot biomass alone. Increasing drought intensity significantly increased the plant root‐to‐shoot ratio (Figure 3A, Table 1, main effect of Drought), indicating that water‐stressed plants invested more resources to potentially increase water uptake. Plant inoculation with all treatments showed a similar trend in increasing root‐to‐shoot ratio. However, plants inoculated with the AcdS^−^ mutant exhibited even greater resource allocation towards root production compared with both uninoculated plants and those inoculated with wild‐type bacteria (Figure 3A, Table 1, main effect of Bacterial treatments, and Bacterial treatments × Drought interaction).

### 3.3 Water Consumption in Plants

Drought treatments significantly altered water consumption in plants (percentage of the water consumed by the plant to total irrigation water) (Figure 3B). In all treatments, almost all of the irrigated water was consumed by plants (mostly via transpiration) under more severe drought intensities. Bacterial inoculation significantly altered plant water consumption (Figure 3B) (Table 1, main effect of Drought, Bacterial treatments, and Drought × Bacteria interaction). The presence of the ACC deaminase gene in the plant‐associated bacteria (Wild‐type treatment) led to the uptake of all available water regardless of the drought level. These results are in line with the observed high stomatal opening ratio (Figure 3C) and high shoot dry weight, especially in well‐watered and mild drought conditions (Figure 2B). In contrast, uninoculated plants and those inoculated with the AcdS^−^ showed significantly (*F*_2, 54_ = 16.04, *p* < 0.001) less water consumption within the drought gradient.

### 3.4 Relative Stomatal Opening

In all bacterial treatments, the ratio of open stomata to total stomata (relative stomatal opening) significantly decreased with increasing drought intensity (Figure 3C, Table 1, the main effect of Drought). In all of the drought treatments, *A. thaliana* inoculated with *Pseudomonas putida* UW4 wild‐type bacteria (WT) had a significantly greater stomatal opening ratio than uninoculated or mutant‐inoculated plants (Figure 3C, Table 1, the main effect of Bacterial treatments).

### 3.5 WUE

Drought intensities significantly altered WUE (biomass per water use) in *A. thaliana* plants (Table 1, main effect of Drought). Decreased stomatal opening with drought would be expected to decrease leaf‐interior CO_2_, which would bring more CO_2_ into the leaf relative to transpiration, increasing WUE. This was true for the uninoculated plants and those with the AcdS^−^ mutant bacteria, both of which showed increasing WUE with drought (Figure 3D). Although plants inoculated with the wild‐type, ACC‐consuming strain also decreased stomatal opening somewhat with drought, this was not enough to prevent severe damage (Figure 1B), so WUE decreased with drought. The presence of the ACC deaminase gene in the WT bacterial strain led to a significant decrease in WUE (with reductions of up to 2.5‐fold observed at low watering frequencies) in drought‐stressed plants, in contrast to the AcdS^−^ mutant (Figure 3D, Table 1, the main effect of Bacterial treatments).

### 3.6 δ^13^ *C* Values

Shoot δ^13^ *C* values were more negative in plants inoculated with the wild‐type bacteria than in plants treated with the AcdS^−^ mutant (Figure 3E) (Table 1, main effect of Bacterial treatments). Increasing drought intensity generally led to more positive δ^13^ *C* values in shoots, as expected with decreased stomatal opening (Figure 3C, Table 1, the main effect of relative stomatal opening). However, the AcdS^−^inoculated plants showed an apparent, unexplained decrease in δ^13^ *C* values across the most extreme (D6) drought treatments.

## 4 Discussion

Drought is a major threat to global food security and causes severe yield reductions (Boyer and Westgate 2004; Mehrabi and Ramankutty 2019). Changes in precipitation patterns associated with climate change are causing longer and drier summers and more severe drought periods (Ferrari et al. 2010; Polade et al. 2017), calling for urgent mitigation strategies to ensure food security. One possible strategy is to harness the soil microbiome to modify the default water‐use strategies of crop plants. For example, when water is a limiting factor, manipulation by microbes might be used to improve plant WUE. This strategy may help complement plant genotypes that have been selected for other traits such as those associated with high yield. Plant strategies to cope with drought are well described and can be used to steer plant breeding programs (Basu et al. 2016). However, given the constraints associated with traditional breeding methods and regulatory difficulties associated with targeted genetic‐engineering approaches in many parts of the world, developing new, drought‐resistant, and water‐wise cultivars may be too tedious or too expensive for widespread application.

In this study, we demonstrate that microbial genes are so intertwined with plant stress responses that they can be used to rewire a plant's water‐use strategy without the need for mutations in the plant genome. Specifically, we show that differences in a single gene in a plant‐associated microorganism can completely shift the plant water management strategy and drought tolerance of the plant.

Relative to uninoculated plants or plants inoculated with the AcdS^−^, plants inoculated with the wild‐type bacteria, ACC‐consuming strain, had more open stomata (Figure 3C), resulting in greater water consumption (Figure 3B), and lower δ^13^ *C* values, (Figure 3E), all consistent with lower WUE and a water‐spender plant strategy. Under well‐watered conditions, this strategy led to greater biomass production and even greater WUE (Figure 3D) than the other bacterial treatments. Under severe drought, however, plants associated with wild‐type bacteria (WT) had significantly reduced WUE compared with plants inoculated with the AcdS^−^ mutant. With longer watering intervals, they ran out of water, with drastic effects on growth (Figures 1 and 2). In the absence of manipulation by the ACC‐consuming wild‐type bacteria, the plant followed a somewhat more conservative strategy sacrificing some growth under well‐watered conditions but improving survival under drought. WUE and δ^13^ *C* were consistent in most drought levels. There was, nonetheless, an unexpected decrease in δ^13^ *C* values in AcdS^−^inoculated plants during the most extreme (D6) drought treatments, which may stem from heightened stress intensity, and therefore, surpassing the plants' physiological adaptation potential.

We observed a water‐wasting phenotype imposed by wild‐type bacterial inoculation, even relative to uninoculated controls. Deleting the bacterial gene encoding for the enzyme ACC deaminase allowed the plant to implement its own somewhat more conservative water‐saving strategy (Figure 3B,D). Uninoculated plants often had intermediate phenotypes (Figures 1, 2, 3). The effect of the introduced bacterial mutation also had a strong impact on the plant's ability to cope with drought. As long as the water was not limiting, all treatments performed well, with wild‐type bacteria (WT) even improving plant growth (Figure 2B). As ACC deaminase activity is often found in beneficial bacteria (Glick and Nascimento 2021), this increase is, therefore, expected under high moisture conditions. As drought increased, plant association with wild‐type bacteria (WT) increased mortality, while the association with AcdS^−^ mutant bacteria, which lack the ability to impair ethylene signalling in plants, led to enhanced drought resistance (or avoidance). This finding suggests the importance of ethylene as a stress‐resistance hormone and adds to the mounting evidence that ACC deaminase disrupts the plant's ability to cope with abiotic stress by impairing ethylene signalling (Ravanbakhsh et al. 2017; Ravanbakhsh, Kowalchuk, and Jousset 2019).

Association with bacteria producing ACC deaminase, an enzyme reducing plant ethylene levels (Figure 2A) (Ravanbakhsh et al. 2017; Ravanbakhsh, Kowalchuk, and Jousset 2019), led to increased water consumption by keeping more stomata open and fostering water transfer from roots to aerial parts of the plant. Plants inoculated with wild‐type bacteria consumed far more water than the other treatments, especially at moderate drought levels. As drought increased, plant growth rapidly declined in relation to other treatments, with plant death as an ultimate result. In a community context, plants with a more conservative strategy could therefore be considered more cooperative.

The effect of a single mutation in the bacterial gene coding for ACC deaminase, which probably allows bacteria to suppress ethylene levels in plants, completely reversed the pattern seen with wild‐type bacteria. Plants associated with AcdS^−^ mutant bacteria were able to better withstand moderate to severe drought relative to those associated with wild‐type bacteria. In particular, plants reacted more appropriately to drought by closing stomata, shifting resource allocation towards the root system, and reducing water transfer to shoots. Together, these adaptations resulted in a higher WUE and a longer survival under prolonged drought (Figure 3D). Allocation of biomass to the roots is a well‐known strategy, helping plants cope with acute and severe drought (Xu et al. 2015), potentially enhancing water uptake.

The phenotype mediated by the wild‐type bacteria perfectly aligns with the definition of a water spender (Urban et al. 2017). A water‐spender phenotype responds to water deficiency by maximizing the use of the remaining water. Given enough water, this allows plants to maintain carbon dioxide flow and photosynthesis (Basu et al. 2016; Yamori et al. 2020), as evidenced by greater biomass in this study (Figures 1 and 2) when plants inoculated with wild‐type bacteria (WT) (Arndt and Wanek 2002b). The water‐spender strategy could benefit plant communities if enough water is consistently available that rapid growth trumps WUE. In mixed communities, individual spenders might benefit at the expense of their more conservative neighbours. However, the water‐spender strategy can rapidly become lethal (in a genetically uniform plant community) when water scarcity persists. After wasting all available water, plants end up with conditions of even greater water deficiency, ultimately leading to death (Schwalm et al. 2017). Our observations were fully in line with this scenario.

We built a minimal holobiont model composed of one plant and one bacterial strain, allowing us to use a reductionist approach to place a model plant regulatory cascade (ethylene signalling) in holobiont context. While, in natural systems and at the full holobiont scale, complex interspecific interactions play a significant role (Berendsen et al. 2012; Leite et al. 2021), this simplified minimal holobiont system can offers several implications by allowing us to disentangle the effects of bacterial gene mutations on plant hormonal balance. First, it demonstrates that drought‐resistant plant phenotypes can be obtained by modification or selection of plant‐associated microorganisms derived from the natural plant microbiome. The first step towards harnessing this potential is to establish and curate the repertoire of bacterial genes affecting the plant phenotype. This is essential in particular in the case of ethylene signalling. Despite mounting evidence that high ethylene levels are essential for plant stress resistance (Arraes et al. 2015), ACC deaminase producers are still often proposed as helping to alleviate stress and promote plant growth (Gamalero and Glick 2022). Numerous microbial activities can impact plant growth and stress tolerance, and it may be the case that observed stress alleviation by ACC deaminase producers is mediated by other mechanisms. Using ethylene‐insensitive mutants in future studies can help disentangle the effects of phenotypic changes mediated via ethylene reduction by bacteria from other microbial metabolites. Several studies demonstrate that the reduction of ethylene levels via plant mutation, chemical treatment, or associated bacteria similarly weakens plant stress tolerance (Ravanbakhsh, Kowalchuk, and Jousset 2019b; Shekhawat et al. 2022; Singh et al. 2015). Specific manipulations of ethylene signalling (or in general all plant hormones) by associated microbes can thus be beneficial under some conditions, but disastrous under others (e.g., the effects of Wild‐type bacteria on plants in Figure 1). We propose that a careful re‐evaluation of most traits implicitly seen as beneficial for plants will be needed to extrapolate microbiome functional gene composition with plant phenotype under different environmental conditions. We acknowledge that we did not measure microbial‐mediated ethylene changes under drought conditions due to the destructive nature of the measurement method. Ethylene production in plants was measured on Day 35 (onset of drought) (Figure 1), and the negative correlation between the wild‐type bacterial strain and ethylene production was shown (Supporting Information S1: Figure S1). Previous research, however, has shown that ethylene pretreatment before stress onset (pretreatment or priming) can create a cascade of ethylene‐mediated stress tolerance throughout the entire plant growth period (Brenya et al. 2023; Mohorović et al. 2023). Further, we call for a rethinking of the use of microorganisms to increase drought resistance in plants. Most studies on plant–microbe interactions are oriented towards maximizing short‐term, individual plant growth (Khalilpour, Mozafari, and Abbaszadeh‐Dahaji 2021; Vurukonda et al. 2016). This may maximize the final yield of an individual plant in a mixed community, but it can be viewed as a selfish strategy, maximizing individual yield at the expense of neighbours. However, in an agricultural setting, field‐level yield can be best maximized by selecting for plant traits that maximize yield at a population level. This implies fostering facilitation or at least reducing competition, as exemplified by the dwarf green revolution crops that compete less for light with their neighbours. In a water‐scarcity context, individual plants maximizing WUE (saver phenotypes) potentially benefit neighbouring plants by leaving more water in the soil for future use (Ford Denison, 2019). This potential benefit, however, is contingent on factors such as low soil‐surface evaporation rates, as high evaporation may direct the conserved water directly to the atmosphere. In contrast, water‐waster plant phenotypes behave selfishly and may reduce overall yields due to the tragedy of the commons (Zea‐Cabrera et al. 2006), whereby plants deplete the community's water supply in their struggle to obtain water for themselves. The evolutionary forces steering the prevalence of such strategies can be extended to the holobiont. A bacterial trait that makes an associated plant sacrifice some growth for the benefit of the plant community (to save water) may not be evolutionarily stable, but may perhaps be steered over timescales that are relevant to agricultural production systems (Sinclair, Devi, and Carter 2016). We propose that strategies that improve plant WUE will be important in the development of sustainable agricultural strategies that maximize yield at the population level, without the need for excessive water input.

From an applied perspective, we anticipate that the results of the present study will help guide microbiome management strategies. For instance, bioaugmentation strategies reducing the abundance of ACC deaminase producers in the microbiome and increasing traits known for drought tolerance improvement may help make agriculture more water‐efficient and sustainable. It remains to understand how such an intervention (e.g., removing the ACC deaminase gene) can impact soil microbial communities in addition to the plant drought tolerance. Such an understanding of plant drought tolerance and the role of the microbial community is expected to become increasingly necessary as larger portions of the world experience drought conditions and decreasing aquifer reserves.

## Conflicts of Interest

The authors declare no conflicts of interest.

## Supporting information

Supporting information.

## Data Availability

The data that support the findings of this study are available from the corresponding author upon reasonable request.
