# Supplementary material for: Deletion of ACC Deaminase in Symbionts Converts the Host Plant From Water Waster to Water Saver
Source: Plant Cell Environ. 2024 Nov 7;48(3):1919–31. doi: 10.1111/pce.15265 (PMC11788950; doi:10.1111/pce.15265)
Supplement: Supplementary file 1 — Supporting information. [file PCE-48-1919-s001.docx]

| **Content** |
| --- |
| **Figure S1…..………………………………………………………………………………………..…… 2** |
| **Tables S1-S5………………………………………..……………………………………………..…….. 3** |
| **Text S1…. …………………………………………….……………………………………………..…… 6** |

**Figure S1**: (A) Effects of the mutation in the ACC deaminase gene on bacterial colonization (CFU/g fresh weight root) in the rhizosphere of *Arabidopsis thaliana* Col-0 plants. (B) Correlation between colonization rates of wild-type bacteria harboring the ACC deaminase gene (X-axis) and shoot ethylene concentrations (Y-axis) in *Arabidopsis thaliana* Col-0 plants under drought stress conditions.

**Table S1:** Descriptive statistics comparing the residual analysis of predicted versus observed values for plant parameters in different drought levels in the continuous model. This table summarizes the variation in data and average trends observed in plant parameters (e.g. shoot dry weight, root dry weight, root-to-shoot ratio) across different drought levels, highlighting the overall central tendencies across different drought levels. Mean close to 0: Residuals (predicted vs observed) are centered around zero. Linearity in the relationship between drought levels and plant parameters is confirmed through residual analysis of predicted versus observed values, where an average close to 0 shows a random distribution of residuals.

| Descriptive Statistics | N | Minimum | Maximum | Mean | Std. Deviation |
| --- | --- | --- | --- | --- | --- |
| Shoot Dry weight | 54 | -162.349 | 143.291 | **0.000** | 69.299 |
| Root dry weight | 54 | -162.349 | 143.291 | **0.000** | 69.299 |
| Root-to-shoot ratio | 54 | -0.416 | 0.594 | **0.000** | 0.248 |
| WUE | 54 | -1.346 | 2.938 | **0.000** | 0.835 |
| 13 Carbon | 54 | -26197.099 | 1149.623 | **0.000** | 3658.964 |
| Water consumption | 54 | -0.159 | 0.081 | **0.000** | 0.061 |
| Stomata closing index | 54 | -0.382 | 0.439 | **0.000** | 0.188 |
| Drought Index | 54 | -0.159 | 0.081 | **0.000** | 0.061 |

**Note:** Bold values indicate statistically significant results (p < 0.05).

**Table S2:** Descriptive statistics of residuals, including skewness and kurtosis values, for assessing the normality of residuals in relation to different drought levels. This table summarizes the skewness and kurtosis values of the residuals from the continuous regression analysis benchmarking the plant parameters vs drought levels (which indicate the distribution of residuals). Skewness values close to 0 suggest a symmetric distribution, while values outside the range of ±2 indicate potential skewness. Kurtosis values close to 0 suggest a normal (bell-shaped) distribution, while values outside this range signal a deviation from normality. Residuals centered around zero and a lack of significant skewness or kurtosis confirm that the linearity assumption is met in the relationship between drought levels and plant growth parameters. Significant negative skewness (-3.182) and leptokurtic distribution (kurtosis = 2.35) indicate that δ¹³C values are not normally distributed, leading to the use of a Generalized Linear Model (GLM) to account for the non-normality.

| Descriptive Statistics | N | Skewness | | Kurtosis | |
| --- | --- | --- | --- | --- | --- |
|  | Statistic | Statistic | Std. Error | Statistic | Std. Error |
| Shoot Dry weight | 54 | -0.081 | 0.325 | -0.073 | 0.639 |
| Root dry weight | 54 | 0.713 | 0.325 | 0.137 | 0.639 |
| Root to shoot ratio | 54 | 0.502 | 0.325 | -0.451 | 0.639 |
| WUE | 54 | 1.165 | 0.325 | 1.825 | 0.639 |
| 13 Carbon | 54 | -3.182 | 0.325 | 2.35 | 0.639 |
| Water consumption | 54 | -1.063 | 0.325 | 1.66 | 0.639 |
| Stomata closing index | 54 | 0.333 | 0.325 | -0.56 | 0.639 |
| Drought Index | 54 | -1.063 | 0.325 | 1.66 | 0.639 |

**Note:** Bold values indicate statistically significant results (p < 0.05).

**Table S3**: The table displays the model selection outcomes using Akaike Information Criteria (AIC) for various plant traits, considering three different bacterial treatments (Uninoculated, wildtype ACC deaminase bacteria, and AcdS- mutant bacteria). R² adjusted values and p-values are presented alongside the selected regression models for each trait. The models include linear, quadratic, and cubic forms to best fit the relationships between variables. The traits analyzed include drought index, shoot dry weight (DW), root DW, root-to-shoot ratio, water use efficiency (WUE), **δ^13^C** measurements, water consumption, and stomata closing index. While lower R² values are observed for certain traits, such as shoot dry weight and δ¹³C, these may reflect biological variability and complex interactions within the system. However, overall trends remain significant, with high p-values across most traits, indicating the model effectively captures key relationships between treatments and plant responses. The quadratic and cubic models capture more complex, non-linear relationships, especially for traits like root-to-shoot ratio and WUE.

|  |  | *R^2^* _adj_ | *P* | Model |
| --- | --- | --- | --- | --- |
| Drought index | Uninoculated | 0.58 | **<0.001** | Quadratic |
|  | AcdS- | 0.7 | **<0.001** | Quadratic |
|  | WT | 0.9 | **<0.001** | Quadratic |
| Shoot DW | Uninoculated | 0.1 | 0.104 | Linear |
|  | AcdS- | 0.12 | **0.048** | Linear |
|  | WT | 0.58 | **<0.001** | Linear |
| Root DW | Uninoculated | 0.02 | 0.21 | Linear |
|  | AcdS- | 0.23 | **0.027** | Linear |
|  | WT | 0.72 | **<0.001** | Linear |
| Root-to-shoot ratio | Uninoculated | 0.32 | **<0.001** | Linear |
|  | AcdS- | 0.66 | **<0.001** | Cubic |
|  | WT | 0.36 | **<0.001** | Quadratic |
| WUE | Uninoculated | 0.3 | **<0.001** | Quadratic |
|  | AcdS- | 0.35 | **<0.001** | Quadratic |
|  | WT | 0.57 | **<0.001** | Quadratic |
| **δ^13^C** | Uninoculated | 0.01 | 0.68 | Quadratic |
|  | AcdS- | 0.31 | **0.013** | Quadratic |
|  | WT | 0.46 | **<0.001** | Quadratic |
| Water consumption | Uninoculated | 0.98 | **<0.001** | Quadratic |
|  | AcdS- | 0.65 | **<0.001** | Quadratic |
|  | WT | 0.1 | 0.06 | Linear |
| Stomata closing index | Uninoculated | 0.87 | **<0.001** | Linear |
|  | AcdS- | 0.48 | **<0.001** | Linear |
|  | WT | 0.43 | **<0.001** | Linear |

**Note:** Bold values indicate statistically significant results (p < 0.05).

**Table S4**: ANOVA table summarizing the interactive effects of categorical variables—drought intensity and bacterial inoculation (uninoculated, WT bacteria interfering with ethylene signaling, or their isogenic AcdS- mutant)—on various facets of plant development under an increasing drought gradient. This analysis investigates the impact of discrete levels of drought and distinct bacterial treatments on plant responses.

| **Dependent variable** | **Effects on parameter** | **Df** | **F-value** | **p-value** |
| --- | --- | --- | --- | --- |
| **Shoot dry weight** | Drought | 2 | 5.87 | **0.001** |
|  | Bacteria | 5 | 2.79 | 0.07 |
|  | Drought x Bacteria | 10 | 2.72 | **0. 001** |
|  | Error | 48 |  |  |
| **Water use efficiency** | Bacteria | 2 | 3.25 | 0.47 |
|  | Drought | 5 | 3.67 | 0.06 |
|  | Drought x Bacteria | 10 | 1.9 | 0.09 |
|  | Error | 48 |  |  |
| **δ^13^C** | Bacteria | 2 | 4.76 | **0.04** |
|  | Drought | 5 | 4.2 | **<0.001** |
|  | Drought x Bacteria | 10 | 11.35 | **<0.001** |
|  | Error | 48 |  |  |

**Note:** Bold values indicate statistically significant results (p < 0.05).

**Table S5**: Post-hoc Comparisons of the effects of drought intensity and bacterial inoculation (uninoculated (ctrl), WT bacteria interfering with ethylene signaling, or their isogenic AcdS- mutant) on various facets of plant development under an increasing drought gradient. The analyzed traits include Shoot Dry Weight (SDW), Root Dry Weight (RDW), Root/Shoot Ratio, Water Consumption, Stomata Index, Water Use Efficiency (WUE), and 13C Index. Post-hoc results are denoted with superscript letters (a, b, c). A similar letter has no significant differences according to the Tukey posthoc test.

|  | SDW | RDW | Root/shoot | Water  consumption | Stomata index | WUE | 13C index |
| --- | --- | --- | --- | --- | --- | --- | --- |
| WT | 167.42a | 40.55c | 0.24c | 251.13a | 0.34b | 0.96b | -33.04c |
| Ctrl | 178.50a | 59.07b | 0.35b | 268.39a | 0.34b | 1.08b | -32.22b |
| AcdS- | 193.53a | 109.81a | 0.66a | 272.42a | 0.48a | 1.71a | -30.00a |

Values in the table are denoted with letters (a, b, c) to indicate significant differences, with significant results bolded for emphasis

**Text S1:** Calculation of water content and designing the watering strategy

The field capacity (FC) of soil is defined as the highest water content that a specific soil can hold against gravitational forces after drainage of excess water and practical cease of downward movement of soil water. In order to appropriately choose the drought levels applied to the plants, we estimated the FC of the soil in the laboratory. We determined the soil water content at FC by saturating oven-dried soil samples with a weight of 120 g, equal to the amount of soil in the plant pots used for the experiment, with water for 24 hours. The pots were covered with plastic foil to avoid evaporation. After saturation, the pots were placed in a petri-dish with a paper base to absorb the gravitationally draining excess water for 72 hours. This amount of time represents the usual period for excess soil water to drain off. The amount of water represents 100% FC. The amount of soil water (SW) at FC was calculated as the weight loss while oven-drying for 24 hours at 105 ℃ using the following formula: SW = Wet Weight (g) – Dry Weight (g). Our soil water at 100% FC was on average 28.75 g. We considered 80% of this water as a control for well-water treatment in the experiment. The rationale behind choosing the water volume (instead of weighting the pots) was to address the complexity of variations in pot biomass due to the effects of bacterial treatments on plant biomass. Using pot weight without considering the biomass variation could be misleading, , especially when the plant biomass is a significant fraction of the total weight. For instance, in some samples, the plant weight exceeded the weight of the soil in the pots. Instead, we calculated the field capacity (FC) of the soil and used 80% of the FC as the criterion for well-watered conditions. We chose the 80% FC for the well-watered control condition based on two criteria: 1) The soil materials were a mix of potting soil (Potgrond Holland B.V., NL, medium size with a mixture of sand, silt, and organic matter), sand (0.1-0.3 mm, classified as medium to coarse sand), and perlite in a 1:1:1 ratio. This combination created a final soil material with medium size and a sandy loam texture, allowing the soil to drain properly (and relatively fast) and dry within 12 hours. Typically, plants start to experience drought stress when the soil moisture level drops below 50-60% of FC. In our soil materials, the soil moisture level drops below 50% of FC after 12 hours (determined by measuring the soil moisture over three days). 2). Administering 23 mL of water twice per day (instead of 46 mL once per day) ensured that the soil remained at the desired field capacity (FC) without experiencing significant fluctuations in moisture content, drying out too much (due to growth chamber ventilation), or becoming overly saturated. We kept the soil water of well-watered control plants (D0) at this water level by adding 23 mL filtered and deionized water (Milli-Q® water) every 12 hours. Drought levels were determined by calculating the respective amount of soil water at 85%, 65%, 55%, 45%, 25%, and 5% of FC (Table 1). Water availability was manipulated by spacing the water application of 23 mL Milli-Q® water per plant every 24, 36, and 48 h for mild drought treatments (D1, D2, and D3, respectively), and every 3, 4, and 18 days for severe drought treatments (D4, D5, and D6, respectively) 1. It is important to note that differences in water use among replicates at each water level could have resulted in variations in soil water content, similar to how two field plots receiving the same amount of rainfall might have different soil water content. Consequently, our approach is more relevant for simulating drought conditions in the field but may be less precise in determining the exact relationship between plant measurements and soil water content.
